# Distributions and sources of traditional and emerging per- and polyfluoroalkyl substances among multiple environmental media in the Qiantang River watershed, China†

**DOI:** 10.1039/d2ra02385g

**Published:** 2022-08-02

**Authors:** Zhengzheng Liu, Jingqing Zhou, Yalu Xu, Jiafeng Lu, Jinyuan Chen, Jing Wang

**Affiliations:** College of Environment, Zhejiang University of Technology Hangzhou China cjy1128@zjut.edu.cn; Zhejiang Ecological and Environmental Monitoring Center, Zhejiang Key Laboratory of Ecological and Environmental Monitoring, Forewarning and Quality Control Hangzhou China

## Abstract

The presence of per- and polyfluoroalkyl substances (PFASs) in the food chain poses a threat to human health. Water and the atmosphere are the major transport pathways for PFASs in the environment, while water, soil and sediment are sinks. Herein, the concentrations and distributions of traditional and emerging PFASs in multi-environmental media samples in the Qiantang River watershed were comprehensively investigated. Twenty-five PFASs, including seven emerging PFASs, were identified. The concentrations in water, soil, sediment and PM_2.5_ ranged from 3.58 to 786 ng L^−1^, 0.72 to 12.3 ng g^−1^, 0.73 to 6.60 ng g^−1^, and 93.9 to 255 pg m^−3^, respectively, with mean concentrations of 149 ng L^−1^, 4.70 ng g^−1^, 4.31 ng g^−1^, and 156 pg m^−3^. Perfluorooctanoic acid (PFOA) was the dominant contaminant in water, soil, and sediment, and perfluoropentanoic acid (PFPeA) was the dominant contaminant in PM_2.5_. Hexafluoropropylene oxide-dimer acid (HFPO-DA) and 6 : 2 chlorinated polyfluorinated ether sulfonate (6 : 2 Cl-PFESA), as substitutes for PFOA and perfluorooctane sulfonate (PFOS), were detected, indicating the gradual replacement of traditional PFOA and PFOS in this area. Perfluoro-3,6-dioxaheptanoic acid (NFDHA), as a component of the aqueous film-forming foam FN-3, was first detected in this area. Short-chain PFASs were mainly distributed in water and PM_2.5_, while long-chain PFASs were distributed in the solid phase, such as soil, sediment, and PM_2.5_. Based on principal component analysis (PCA), the major PFAS sources were emulsifiers from fluorine polymerization and surface-active agents from the textile, papermaking, leather, and other industries. In addition, correlation analysis showed that water was the main source and transport pathway of short-chain perfluoroalkyl carboxylic acid (PFCA), HFPO-DA, and NFDHA in this area, while the atmosphere combined with PM_2.5_ was the main transport pathway for both short- and long-chain PFCAs, PFOS, and 6 : 2 Cl-PFESA.

## Abstract 2

The presence of per- and polyfluoroalkyl substances (PFASs) in the food chain poses a threat to human health.

## Graphical abstract

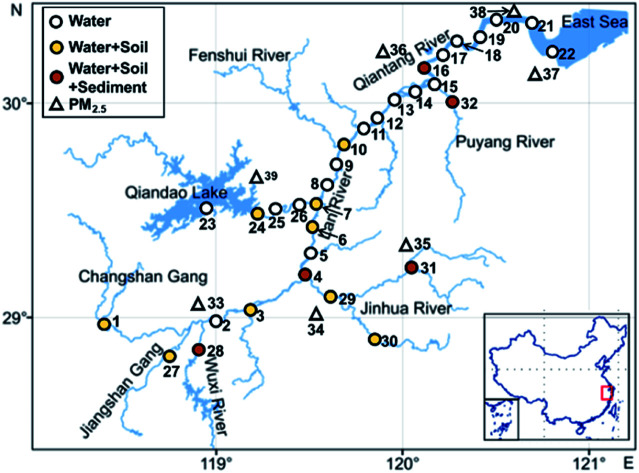

## Introduction

Per- and polyfluoroalkyl substances (PFASs) have unique surface activity (such as water and oil repellency, thermal and acid resistance) and high stability, and are widely used in the production of consumer goods (such as textiles, paper, non-stick cookware, carpets, and detergents), industrial manufacturing (such as metal coatings, fire-fighting foams, electronics, and photography), *etc.*^1–4^ PFASs are persistent and bioaccumulative and enter the environment through the atmosphere, water, soil and sediment diffusion and long-distance transport.^2,5–8^ The environmental problems caused by PFASs have gradually attracted increasing attention. With the restricted use of perfluorooctanoic acid (PFOA) and perfluorooctane sulfonate (PFOS), some emerging PFAS substitutes, such as perfluoroalkyl ether carboxylic acids (PFECAs) and perfluoroalkyl ether sulfonic acids (PFESAs), have been introduced to the market.^9–12^ Hexafluoropropylene oxide-dimer acid (HFPO-DA, denoted as GenX) and sodium 4,8-dioxa-3*H*-perfluorononanoate (ADONA) are alternatives for long-chain PFASs, and have shorter half-lives in humans and biota.^11,13–15^ However, studies have shown that HFPO-DA and ADONA exhibit biotoxicity similar to that of PFOA and have the potential to bioaccumulate.^11^ In the chromium plating industry, 6 : 2 chlorinated polyfluorinated ether sulfonate (6 : 2 Cl-PFESA, F-53B) has been used in China for decades. F-53B is an endocrine disruptor that can interfere with the thyroid system.^11^

In recent years, emerging PFASs have been frequently detected in the environment. For example, Heydebreck *et al.* found that both traditional PFASs and their substitute HFPO-DA were detected in the water of the Elbe and Rhine Rivers in Germany, the Rhine Meuse delta in the Netherlands, and the Xiaoqing River in China. The main pollutant in the Xiaoqing River in China was PFOA, while the main pollutant in the Scheur River in Germany was HFPO-DA.^16^ Ma *et al.* found that PFOA was the main pollutant in the topsoil of Tianjin, but the concentration of 6 : 2 Cl-PFESA (a PFOS substitute) was higher than that of PFOS.^9^ In Asian atmospheric particulate matter, the main pollutants were perfluorinated carboxylic acids (PFCAs), which are mainly adsorbed on fine particulate matter (PM_2.5_). Land use type, such as urban and costal-dusty, is the main factor affecting the distribution of PFASs in particulate matter, as they can affect the size and surface area of the particles.^17^ Different from the distribution characteristics of PFASs in water, soil, and particulate matter in the above regions, Ali *et al.* found that PFOS and PFBA showed the highest concentrations among PFASs in seafloor sediments in the eastern Red Sea and that PFOS and 6 : 2 fluorotelomer sulfonate (6 : 2 FTS, a substitute for PFOS) accumulated in edible fish in the Red Sea.^18^ However, the research on PFASs in the environment is mainly focused on single environmental media and dual environmental media; only a few studies have investigated PFASs in multiple environmental media, and most of them are focused on traditional PFASs. Sammut *et al.* found that in Malta, the distributions of PFASs in surface water and rainwater were correlated with the distributions of PFASs in environmental media such as sediment, soil, and groundwater; the PFASs in sediment originated from surface runoff and precipitation, and the PFASs in soil and sediment eventually entered the groundwater.^19,20^ The water environment and atmosphere of a watershed are important pollution sources and transport media for PFASs.^21,22^ Water, soil and sediment are important sinks for PFASs in the environment.^23–27^ Therefore, the study of the distribution of PFASs in different environmental media in the same watershed, especially the distribution of emerging PFASs in different environmental media, is of great value for understanding the transport and fate of PFASs in the environment.

The Qiantang River is located on the southeast coast of China. In this study, we sought to comprehensively characterize the traditional and emerging PFASs in multiple environmental media of the Qiantang River watershed, including the surface water, soil, sediment, and PM_2.5_. Principal component analysis (PCA) was used to identify the potential sources of PFASs in the watershed and to explore the correlations and possible fates of PFASs in different environmental media.

## Experimental section

### Materials and reagents

All the standard and internal standard (IS) formulations of the studied compounds were purchased from Wellington Laboratories (Canada). The eleven PFCAs included perfluorobutanoic acid (PFBA), PFPeA, perfluorohexanoic acid (PFHxA), perfluoroheptanoic acid (PFHpA), PFOA, perfluorononanoic acid (PFNA), perfluorodecanoic acid (PFDA), perfluoroundecanoic acid (PFUnA), perfluorododecanoic acid (PFDoA), perfluorotridecanoic acid (PFTrA), and perfluorotetradecanoic acid (PFTeA). The five perfluorinated sulfonic acids (PFSAs) included perfluorobutane sulfonate (PFBS), perfluorohexane sulfonate (PFHxS), perfluoroheptane sulfonate (PFHpS), PFOS, and perfluorodecane sulfonate (PFDS). The two precursors included *N*-methylperfluoro octanesulfonamidoacetic acid (*N*-MeFOSAA) and *N*-ethylperfluorooctane sulfonamidoacetic acid (*N*-EtFOSAA), and the eight perfluoropolyether substitutes included HFPO-DA, ADONA, 6 : 2 Cl-PFESA, 8 : 2 chlorinated perfluoroether sulfonic acid (8 : 2 Cl-PFESA), perfluoro-methoxypropionic acid (PFMPA), perfluoro-4-methoxybutanoic acid (PFMBA), perfluoro-3,6-dioxaheptanoic acid (NFDHA), and perfluoro-2-ethoxyethane sulfonate (PFEESA). ISs included ^13^C_4_-PFBA, ^13^C_2_-PFHxA, ^13^C_4_-PFOA, ^13^C_5_-PFNA, ^13^C_2_-PFDA, ^13^C_2_-PFUdA, ^13^C_2_-PFDoA, ^18^O_2_-PFHxS, ^13^C_4_-PFOS, and D_3_-*N*-MeFOSAA.

Methanol, acetonitrile, and formic acid were all of LC-MS grade from Thermo Fisher Scientific (USA). Ammonium acetate and ammonia was obtained from Merck Millipore (Darmstadt, Germany). Dichloromethane and acetone of chromatographic grade were provided by TEDIA company (USA); Waters Oasis® WAX solid-phase extraction (SPE) cartridges (500 mg, 6 mL) were obtained from Waters (USA).

### Sample collection

**Fig. 1:**
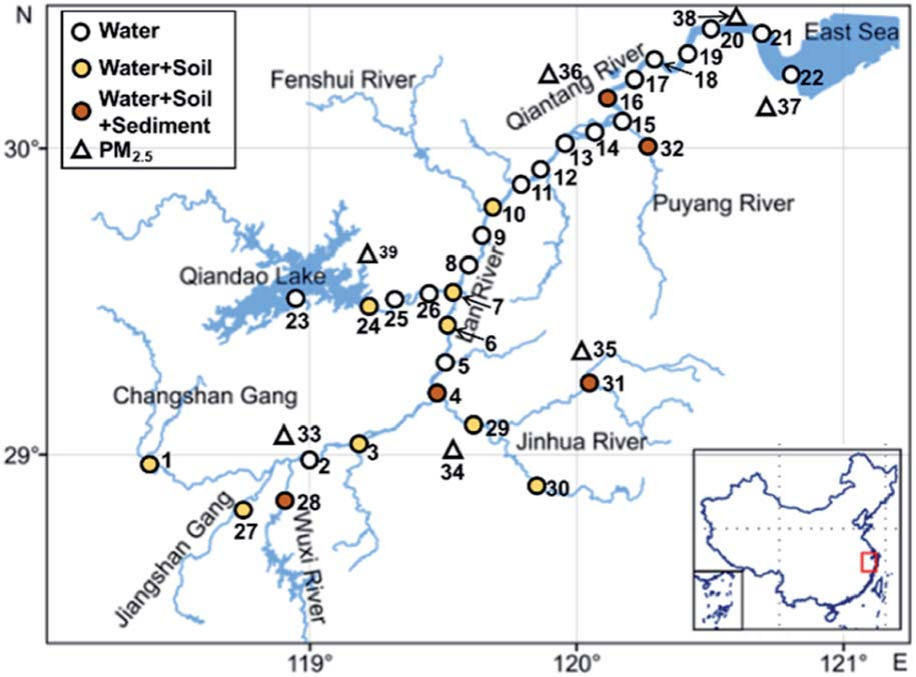
Map of the research area and the sampling sites along the Qiantang Rivers watershed.

All samples were collected in May 2020. A total of 32 surface water sampling sites were set up (Fig. 1) to collect river surface water samples. Coastal topsoil samples were collected at 14 of these sampling sites, and sediment samples were collected at 5 of these sites. PM_2.5_ sampling sites were set up in 7 cities along the Qiantang River watershed to collect PM_2.5_ once every 3 days for a continuous month.

### Sample preparation

#### Water extraction

PFASs were extracted from water samples using the WAX cartridges based on the ISO 25101 method.^28^ The cartridge was pre-conditioned with 4 mL of 0.5% (v/v) ammonia/methanol, methanol, and ultra-pure water. A volume of 1 L of water (pH ≈ 3) spiked with 5 ng of IS was loaded into the cartridge. After nitrogen was passed through for 15 min, the cartridge was washed with 4 mL of methanol and eluted with 4 mL of 0.5% (v/v) ammonia/methanol twice. The extract was concentrated by evaporating to a total of 500 μL using a nitrogen/bath evaporator and subsequently diluted to 1 mL with 2 mmol L^−1^ NH_4_Ac in water.

#### Sediment/soil extraction

The methanol extraction previously described by Li *et al.* was used here.^29^ Two grams of soil or sediment samples was spiked with 5 ng of IS. The soil or sediment was then extracted three times using 5 mL of methanol, vortexed, sonicated for 15 min, and centrifuged at 3000 rpm for 10 min. The extracts were then brought to 1 L with the addition of 18 MΩ ultra-pure water and finally treated as the water samples described above.

#### PM 2.5 filter extraction

A procedure established previously with some modifications was used.^30^ Briefly, each filtered sample was extracted by dichloromethane/acetone 2 : 1 (v/v) using accelerated solvent extraction (ASE) with 3 circulations. The temperature was 100 °C, and the pressure was 15 MPa. The volume was then reduced to 30 mL under nitrogen and brought to 1 L with the addition of 18 MΩ ultra-pure water and finally treated as the water samples described above.

### Sample analysis

All the samples were analysed using ultra-performance liquid chromatography (UPLC) combined with a 6500 Qtrap MS system equipped with an electrospray ionization (ESI) source (AB SCIEX, USA). The optimized ESI operating parameters for negative mode were as follows: ion spray (IS), −4500 V; curtain gas (CUR), 35 psi; temperature (TEMP), 400 °C; gas 1 (GS1), 55 psi; and gas 2 (GS2), 55 psi. PFASs were separated with a BEH C18 column (130 Å, 1.7 μm, 2.1 × 50 mm). The eluent system consisted of (A) 2 mmol L^−1^ NH_4_Ac in water and (B) methanol. The gradient was programmed as follows: 0–0.5 min, 5% solvent B; 1.5 min, 45% solvent B; 6–8 min, 95% solvent B; and 8.1–10 min, 5% solvent B. The flow rate was maintained at 0.3 mL min^−1^ throughout the run, and the sample volume injected was 5 μL.

### Quality assurance and control

The internal standard method was used for quantification. The concentration range of the calibration curve was 0.01–10.0 μg L^−1^, the correlation coefficient (*r*) of the calibration curve of PFASs was between 0.9911 and 0.9998, the recovery rate was between 60% and 140%, and the relative standard deviations were all below 20%, as shown in Table S1.† The instrumental lower limit of detection (LLOD) and lower limit of quantitation (LLOQ) were determined based on the 3-fold signal-to-noise ratio and the 10-fold signal-to-noise ratio, respectively, and then converted to the corresponding methodological LLOD and LLOQ according to the quantity of samples used, as shown in Table S1.† Sampling containers and instrument accessories made of polytetrafluoroethylene were avoided during sample analysis. The concentration of PFASs in the blank was lower than the methodological LLOQ.

## Results and discussion

### Levels and composition characteristics of PFASs in the environment of the Qiantang River watershed

**Fig. 2:**
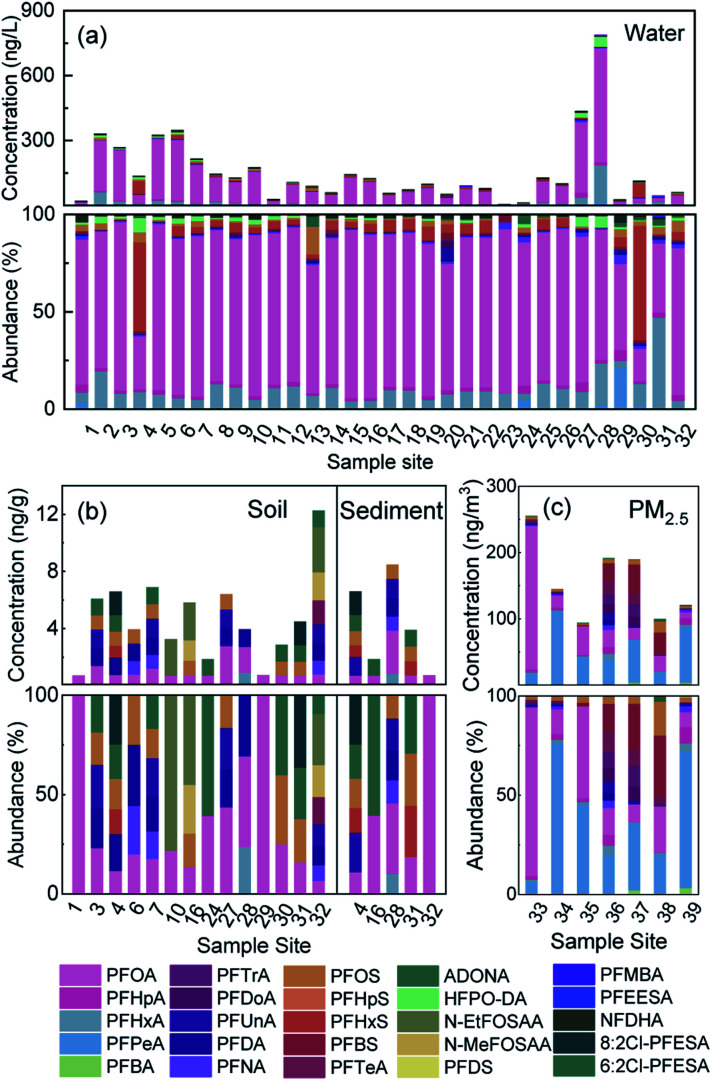
Concentrations and abundances of PFASs in different environmental matrices, (a) concentrations and abundances of PFASs in water. (b) Concentrations and abundances of PFASs in soil and sediment. (c) Concentrations and abundances of PFASs in PM_2.5_.

The concentration levels and detection frequencies (DFs) of all PFASs measured in the multiple media are shown in Table S2.† Among the 25 PFASs detected in the watershed, 18 were traditional, and 7 were emerging. The concentrations and proportions of these PFASs are shown in Fig. 2. The concentrations of total PFASs (ΣPFASs) in the water samples ranged from 3.58 to 786 ng L^−1^ (149 ng L^−1^ on average), with PFOA and PFHxA accounting for the highest proportions, 73.1% and 10.9%, respectively, which is similar to the results of previous studies on the water in this watershed (dominant PFOA was 58.1% and PFHxA was 18.8%).^31^ The concentrations of ΣPFASs in the soil samples ranged from 0.72 to 12.3 ng g^−1^ (4.70 ng g^−1^ on average), with PFOA and PFDA accounting for the highest proportions, 22.3% and 12.9%, respectively. The concentrations of ΣPFASs in the sediment samples ranged from 0.73 to 6.60 ng g^−1^ (4.31 ng g^−1^ on average), with PFOA and 6 : 2 Cl-PFESA accounting for the highest proportions, 27.4% and 15.8%, respectively. The concentrations of ΣPFASs in PM_2.5_ samples ranged from 93.9 to 255 pg m^−3^ (156 pg m^−3^ on average), with PFPeA and PFOA accounting for the highest proportions, 34.4% and 32.2%, respectively.

Studies have shown that the concentrations of ΣPFASs in surface water, drinking water, soil, and sediment in China range from 7.0 to 489 ng L^−1^, 4.49 to 174.93 ng L^−1^, 244 to 13 564 pg g^−1^, and 0.086 to 5.79 ng g^−1^, respectively.^29,32–34^ The concentration level of ΣPFASs in the environment of the Qiantang River watershed is comparable to the overall level of ΣPFASs in China.

The concentration of ΣPFASs in the atmosphere (including air and particulate matter) in China is within the range of 3.4–34 pg m^−3^.^21^ The concentration of PFASs in the PM_2.5_ in Beijing, China, is within the range of 2.3–290 pg m^−3^.^35^

The concentration of ΣPFASs in the PM_2.5_ in the Qiantang River watershed is higher than the average level in China, but is consistent with the level of Beijing. PFOA is the main PFAS in the Qiantang River watershed. It is detected in all environmental media and has the highest proportions in water, soil, and sediment. However, in PM_2.5_, the proportion of PFPeA is the highest because some of the PFPeA in PM_2.5_ is derived from the degradation of precursor substances such as 6 : 2 fluorotelomer alcohol (6 : 2 FTOH).^21^

Two of the emerging PFASs, HFPO-DA and NFDHA, were mainly detected in water samples. The highest concentrations of HFPO-DA occurred at sites 28 (47.1 ng L^−1^) and 27 (22.2 ng L^−1^), which was located in a fluorine chemical industry cluster area, and the HFPO-DA in water might be derived from wastewater discharge from fluoropolymer production.^16^ NFDHA is the main component of the light water foam fire extinguishing agent FN-3. It was detected for the first time in this watershed. Hence, FN-3 might be produced and used in the watershed. The PFAS 6 : 2 Cl-PFESA was detected in all environmental media in the watershed; 6 : 2 and 8 : 2 Cl-PFESA are the major and minor components, respectively, of the chromium fog inhibitor F-53B.^36^ The concentration ratio of 6 : 2 and 8 : 2 Cl-PFESA in water was 15.6, which is close to that in F-53B (12.9 ± 2.6), indicating that F-53B might be used in the electroplating industry in the watershed.^37^ It is worth noting that the concentrations of 6 : 2 Cl-PFESA in soil and sediment were comparable to those of PFOS in soil and sediment, respectively. According to the calculation (Table S3†), the partition coefficient of 6 : 2 Cl-PFESA in water and sediment (lg *K*_d_ = 3.04 ng kg^−1^) was greater than that of PFOS (lg *K*_d_ = 2.81 ng kg^−1^), and 6 : 2 Cl-PFESA was more easily adsorbed into soil and sediment. Ma *et al.* also found that the detection rate of 6 : 2 Cl-PFESA was higher than that of PFOS in the soil of Tianjin, China. After 6 : 2 Cl-PFESA enters the environment, it is easily absorbed by biological organisms due to its high bioaccumulative capacity, thus causing harm.^9^

**Fig. 3:**
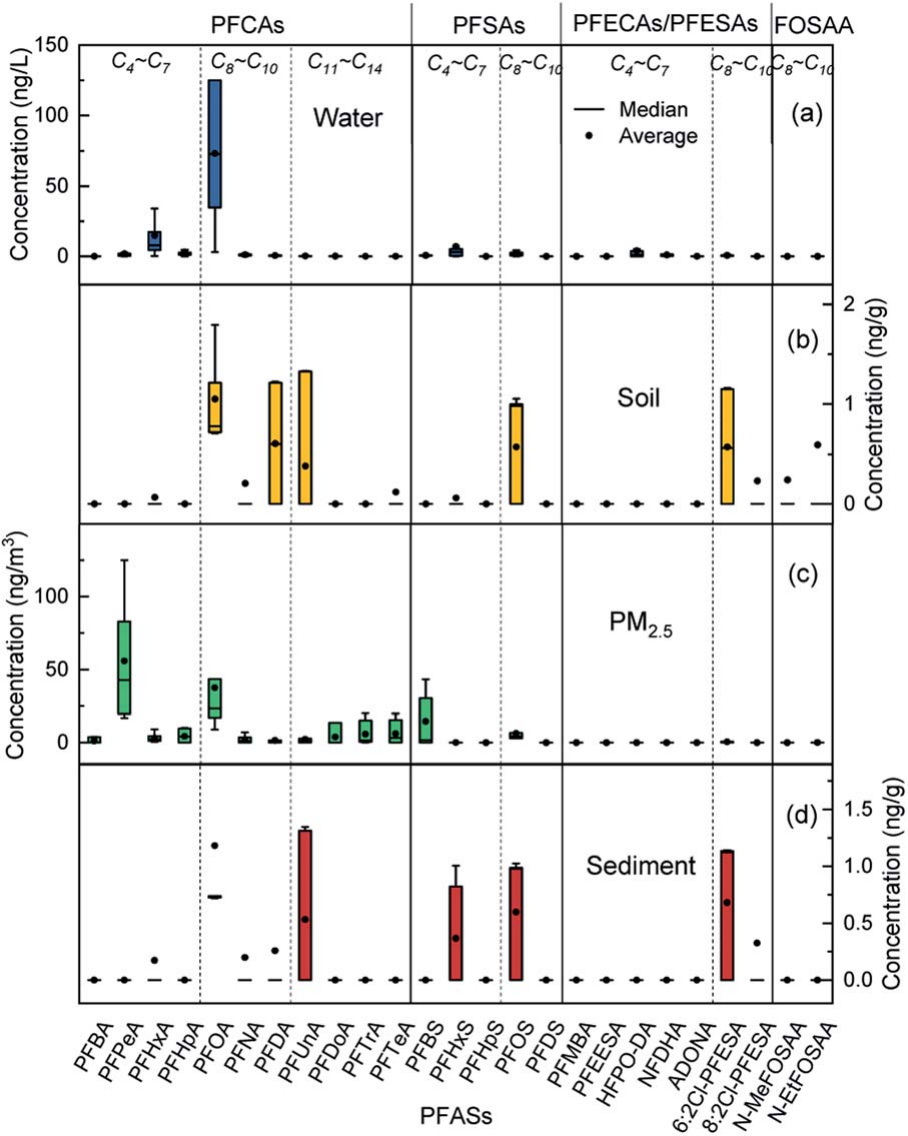
Distribution of PFASs in different environmental media, (a) water, (b) soil, (c) PM_2.5_, (d) sediment.

The distribution of PFASs in different environmental media is shown in Fig. 3. The short-chain PFASs showed higher concentrations in water and PM_2.5_, while the long-chain PFASs showed higher concentrations in soil and sediment. Short-chain PFASs have greater water solubility and higher vapour pressure, so they enter surface water and the atmosphere more easily than long-chain PFASs.^38,39^ Long-chain PFASs are highly hydrophobic and have a high solid–liquid partition coefficient, so they tend to partition into sediment and suspended particles.^40^ Long-chain PFASs also presented a certain distribution in PM_2.5_, indicating that long-chain PFASs entering the atmosphere are more likely to be distributed into atmospheric particulate matter. Precursor substances were more abundant in soil than in sediment, and soil was more susceptible to pollution from point sources such as PFAS manufacturing plants and sewage treatment plants than sediment.

### Spatial distribution characteristics of PFASs in the environment of the Qiantang River watershed

**Fig. 4:**
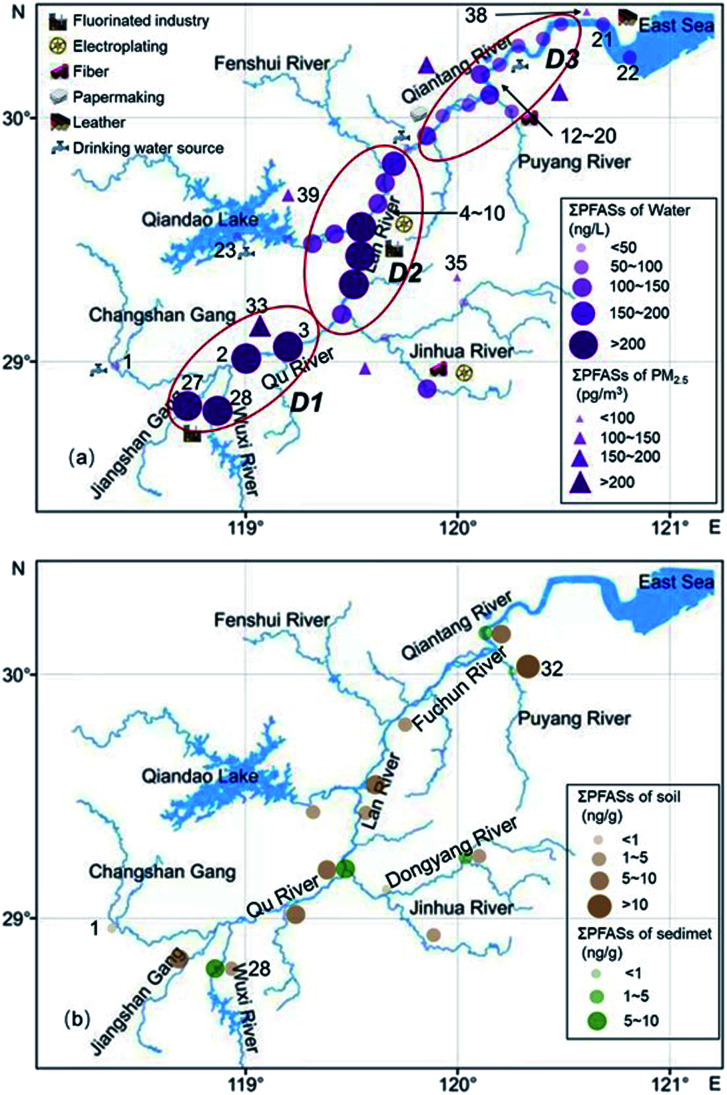
Spatial distribution characteristics of PFASs in the Qiantang River, (a) spatial distribution characteristics of PFASs in water and PM_2.5_. (b) Spatial distribution characteristics of PFASs in soil and sediment.

The distribution of the mass concentration of ΣPFASs in the environment at each site is shown in Fig. 4. The mass concentrations of ΣPFASs were higher in the upper reaches of the Qiantang River watershed than in the lower reaches.

The mass concentration of ΣPFASs in the water was highest at site 28 (786 ng L^−1^) and lowest at site 23 (3.58 ng L^−1^). Area D1 had the highest concentration of ΣPFASs in the entire watershed. PFASs originated from the nearby fluorine chemical industry cluster area.^41^ Area D2 had fewer facilities using fluorine chemicals, as well as textile, nonferrous metal smelting and metal surface treatment industries. Although river branches with low PFAS concentrations flowed into this area, the concentration of ΣPFASs in this area remained at a high level. The ΣPFASs in area D3 ranged from 51.0 to 142 ng L^−1^ (88.5 ng L^−1^ on average). The PFASs composition profiles were similar in this area (from site 13 to site 19), with inputs from upstream PFASs and emissions from the papermaking, food packaging, and film coating industries.^41^ Sites 21 and 22 were located in the estuary. Despite of the dilution effect of seawater, the concentrations of ΣPFASs at sites 21 and 22 were comparable to that in area D3, and the PFASs at sites 21 and 22 originated from the nearby tannery, textile, and chemical fibre industries.

The concentration of ΣPFASs in PM_2.5_ was highest at site 33 (255 pg m^−3^) and lowest at site 35 (93.9 pg m^−3^), which was consistent with the distribution of PFASs in water. The concentration of ΣPFASs in water was lowest near site 39, but the concentration of ΣPFASs in PM_2.5_ near site 39 was higher than that at site 35 or 38. This is mainly because during the sampling period, the dominant wind direction in this area was southeast (Fig. S1†), and the PFASs in the atmosphere at areas D1 and D2 were transported over long distances with air and particulate matter, resulting in an increased concentration of PFASs in PM_2.5_ in this area.^31,42^ There are a large number of textile, chemical fibre, and tannery factories near site 38, but the concentrations of ΣPFASs at this site were relatively low, mainly because the clean air from the ocean (Fig. S1†) reduced the concentration of PFASs in PM_2.5_ in this area.^30^

The distributions of PFASs in soil and sediment were similar to that in water. The concentration of ΣPFASs in soil was highest at site 32 (12.3 ng L^−1^) and lowest at site 1 (0.72 ng L^−1^). The concentration of ΣPFASs in sediment was highest at site 28 (8.48 ng L^−1^) and lowest at site 32 (0.73 ng L^−1^). The soil near site 32 contained relatively high concentrations of two precursors, *N*-MeFOSAA and *N*-EtFOSAA, which are often found in the activated sludge of sewage treatment plants. The soil at site 32 might be affected by the point source discharge of the nearby sewage treatment plant, which needs to be verified by further investigation.

### Source analysis of PFASs in the Qiantang River watershed

**Fig. 5:**
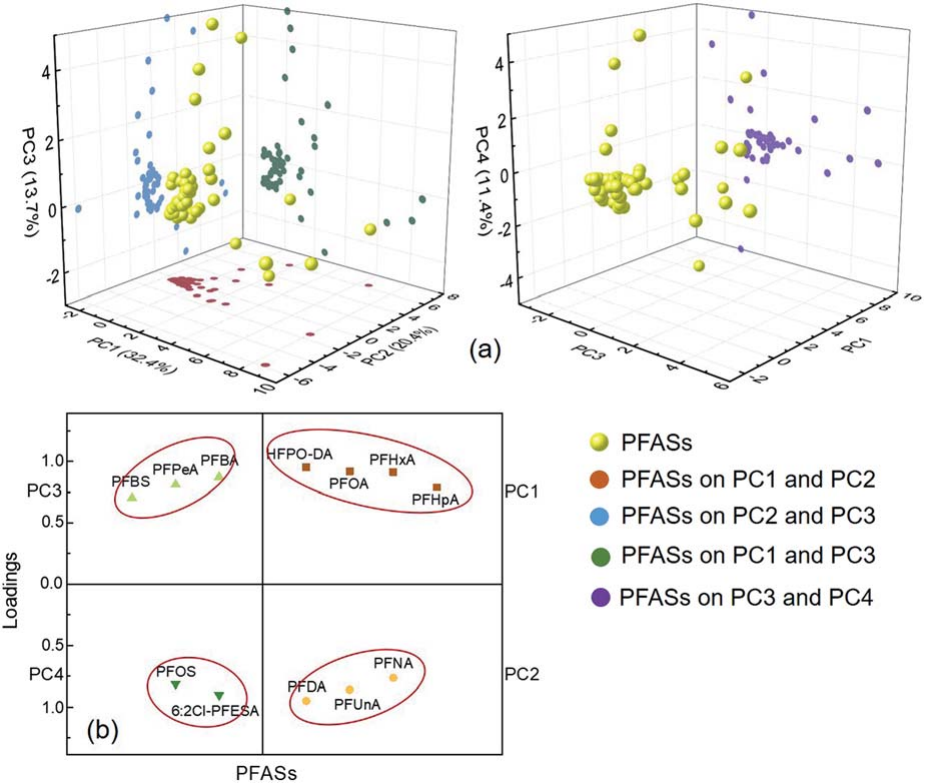
PCA score (a) and loading plots (b) of PFAS patterns in various localities.

PCA is often used to analyse the sources of PFASs in environmental media.^43,44^ In this study, PCA was used to analyse the source of 25 PFASs detected in the watershed environment, and four principal components (PCs) were extracted from the PFASs in the environment of the Qiantang River watershed (Fig. 5(a)). The contribution rates of the four PCs and the loadings of predominant PFASs are shown in Fig. 5(b). The consistencies between the extracted PCs and the actual PFAS emission sources can be determined according to the typical markers of PFASs derived from different sources.^43^ HFPO-DA and PFOA are derived from industrial emulsification of fluoropolymers, flame retardation of textiles, rubber emulsification, food packaging, and paper surface treatment.^2,31,45^ PFHxA is used as a water repellent in the textile, paper, and leather industries, and PFHpA is mainly used as a surfactant.^31,46^ PFNA, PFDA, and PFUnA are mainly derived from the emissions from the production of perfluorocarboxylic acids and intermediates.^2,47^ Short-chain PFASs, such as PFBA, PFPeA, and PFBS, may be derived from the degradation of other PFASs.^32^ PFOS and 6 : 2 Cl-PFESA are mainly used in the petrochemical industry, hardware electroplating, and electronics cleaning.^48^ Therefore, PC1 can be interpreted as the emulsification and surface activation of fluoropolymers, textiles, rubber, paper, and leather; PC2 can be interpreted as the production of PFASs; PC3 can be interpreted as the degradation of PFASs; and PC4 can be interpreted as petrochemical processing, electroplating, and electronics cleaning.

According to the results of this study, the most important source of PFASs in the Qiantang River watershed is from the emulsification and surface activation of fluoropolymers, flame retardants, rubber, textiles, paper, and leather, followed by the production of PFASs and petrochemical processing, electroplating, and electronics cleaning.

### Primary transport pathways of predominant PFASs in the environment of the Qiantang River watershed

**Fig. 6:**
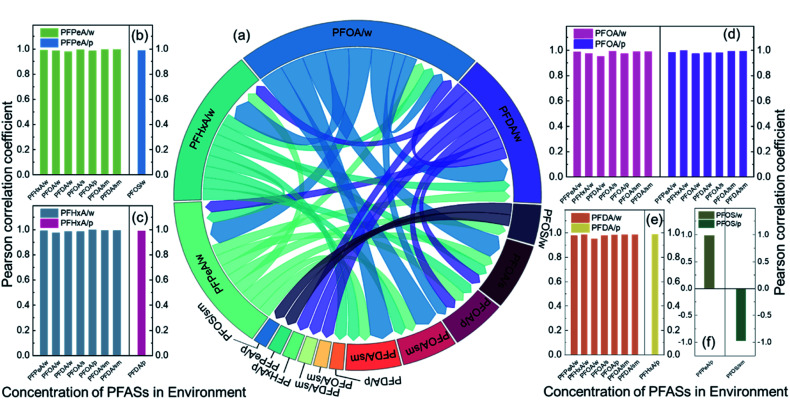
The correlations between different PFASs in multiple environmental media. (a) The relationship between PFASs in multiple environmental media. (b) Pearson correlation coefficients of PFPeA in water and PM_2.5_ with other PFASs. (c) Pearson correlation coefficients of PFHxA in water and PM_2.5_ with other PFASs. (d) Pearson correlation coefficients of PFOA in water and PM_2.5_ with other PFASs. (e) Pearson correlation coefficients of PFDA in water and PM_2.5_ with other PFASs. (f) Pearson correlation coefficients of PFOS in water and PM_2.5_ with other PFASs. “/w” means in water, “/s” means in soil, “/sm” means in sediment, and “/p” means in PM_2.5_.

Water and PM_2.5_ are the two major transmission media for PFASs in the environment. This study separately analysed the correlations of the predominant PFASs in water and PM_2.5_ with the PFASs in different environmental media. Fig. 6(a) shows the correlation between PFASs in different media. Fig. 6(b)–(f) shows the Pearson correlation coefficients of the concentration of each PFAS in water or PM_2.5_ with PFASs in other environmental media (*P* < 0.01 and *P* < 0.05). PFOA in water had significant positive correlations with PFPeA, PFHxA, and PFDA in water and with PFOA in soil, sediment, and PM_2.5_. This finding indicates that PFASs in water are the main source of PFASs in the watershed, with water as the main transmission medium. PFOA in PM_2.5_ had significant positive correlations with PFPeA, PFHxA, PFOA, and PFDA in water and with PFOA and PFDA in sediment, which indicates that PM_2.5_ is an important transmission medium for PFASs. PFPeA in PM_2.5_ had a significant positive correlation with PFOS in water and a significant negative correlation with PFOS in sediment. PFOS/PFOA is often used to determine the potential source of PFASs.^49^ A PFOS/PFOA value exceeding 1.0 indicates the presence of point source pollution of PFOS, while a PFOS/PFOA value below 1.0 indicates that PFOS mainly originates from rainfall. The value of PFOS/PFOA in the Qiantang River watershed was between 0 and 0.21, suggesting that the PFOS in the watershed mainly came from atmospheric deposition. The precursor substances (fluorosulfamido alcohol, *etc.*) volatilize into the atmosphere and are oxidized to form PFOS, which then enters water bodies through atmospheric deposition and is redistributed into the sediment.^10^

**Fig. 7:**
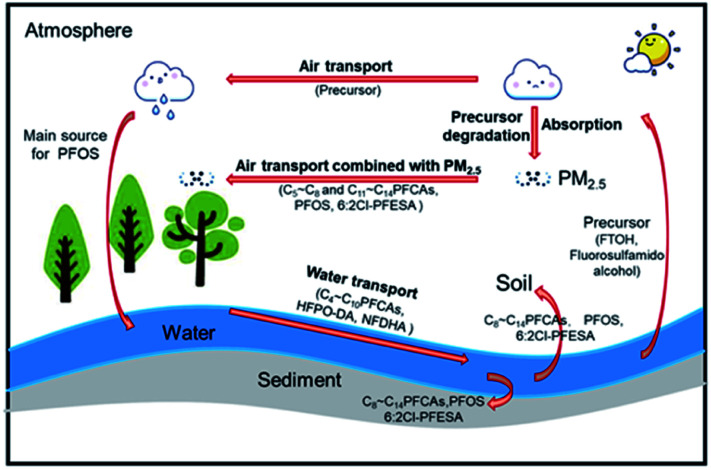
Transport pathways of PFASs in the Qiantang River watershed.

According to this study and the findings of Wang *et al.*, the transport pathways of PFASs in the watershed are speculated, as shown in Fig. 7.^21^ C_4_–C_10_ PFCAs, HFPO-DA, and NFDHA were mainly distributed in water and transported with water. C_8_–C_14_ PFCAs, PFOS, and 6 : 2 Cl-PFESA were distributed into sediment and nearby soil. C_5_–C_8_ and C_11_–C_14_ PFCAs, PFOS, and 6 : 2 Cl-PFESA were adsorbed on the particulate matter and travelled long distances in the atmosphere.

## Conclusions

The results of this study clearly revealed that both traditional and emerging PFASs were detected in the Qiantang River watershed, in which PFOA was the dominant contaminant in water, soil, and sediment, and PFPeA was the dominant contaminant in PM_2.5_. The higher concentration in densely populated areas and industrial parks indicates that human activities greatly affected the occurrence of PFASs in the environment. The occurrence of HFPO-DA and 6 : 2 Cl-PFESA indicated the use of emerging PFAS alternatives. Potential sources of PFAS release in the area were identified with a PCA model, which showed that industrial sources had a primary role in this area, which may cause potential risks associated with PFAS pollution. In addition to transport by water, transport *via* atmospheric particles was an important pathway for volatile PFAS migration, which indicates that it is necessary to investigate the distribution characteristics of volatile PFASs such as FTOH in the atmosphere and airborne particles.

## Conflicts of interest

There are no conflicts to declare.

## Supplementary Material

RA-012-D2RA02385G-s001
