# Supplementary material for: Distributions and sources of traditional and emerging per- and polyfluoroalkyl substances among multiple environmental media in the Qiantang River watershed, China
Source: RSC Adv. 2022 Aug 2;12(33):21247–54. doi: 10.1039/d2ra02385g (PMC9345021; doi:10.1039/d2ra02385g)
Supplement: RA-012-D2RA02385G-s001 [file RA-012-D2RA02385G-s001.pdf]

Electronic Supplementary Material (ESI) for RSC Advances.
This journal is © The Royal Society of Chemistry 2022

Supporting Information

Distributions and sources of traditional and emerging per- and polyfluoroalkyl substances among multiple environmental media in the Qiantang River watershed, China

Zhengzheng Liu, [a] Jingqing Zhou, [b] Yalu Xu, [b] Jiafeng Lu, [b] Jinyuan Chen, *[a] and Jing Wang *[b]

[a.] College of Environment, Zhejiang University of Technology, Hangzhou, China. E-mail: cjy1128@zjut.edu.cn

[b.] Zhejiang Ecological and Environmental Monitoring Center, Zhejiang Ecological and Environmental Monitoring Center, Zhejiang Key Laboratory of Ecological and Environmental Monitoring, Forewarning and Quality Control, Hangzhou, China

TableS1 The abbreviations and structures of PFASs

Compound name	Abbreviation	Structures	Category
Perfluorobutanoic acid	PFBA	$CF_3(CF_2)_2COOH$	T
Perfluoropentanoic acid	PFPeA	$CF_3(CF_2)_3COOH$	T
Perfluorohexanoic acid	PFHxA	$CF_3(CF_2)_4COOH$	T
Perfluoroheptanoic acid	PFHpA	$CF_3(CF_2)_5COOH$	T
Perfluorooctanoic acid	PFOA	$CF_3(CF_2)_6COOH$	T
Perfluorononanoic acid	PFNA	$CF_3(CF_2)_7COOH$	T
Perfluorodecanoic acid	PFDA	$CF_3(CF_2)_8COOH$	T
Perfluoroundecanoic acid	PFUnA	$CF_3(CF_2)_9COOH$	T
Perfluorododecanoic acid	PFDoA	$CF_3(CF_2)_{10}COOH$	T
Perfluorotridecanoic acid	PFTrA	$CF_3(CF_2)_{11}COOH$	T
Perfluorotetradecanoic acid	PFTeA	$CF_3(CF_2)_{12}COOH$	T
Perfluorobutane sulfonate	PFBS	$CF_3(CF_2)_3SO_3H$	T
Perfluorohexane sulfonate	PFHxS	$CF_3(CF_2)_5SO_3H$	T
Perfluoroheptane sulfonate	PFHpS	$CF_3(CF_2)_6SO_3H$	T
Perfluorooctane sulfonate	PFOS	$CF_3(CF_2)_7SO_3H$	T
Perfluorodecane sulfonate	PFDS	$CF_3(CF_2)_9SO_3H$	T
N-methylperfluoro octanesulfonamidoacetic acid	N-MeFOSAA	$CF_3(CF_2)_7SO_2N(CH_3)CH_2CO_2H$	T
N-ethylperfluorooctane sulfonamidoacetic acid	N-EtFOSAA	$CF_3(CF_2)_7SO_2N(CH_2CH_3)CH_2CO_2H$	T
Hexafluoropropylene oxide-dimer acid	HFPO-DA	$CF_3(CF_2)_2$ $OCF(CF_3)COOH$	E
Sodium 4,8-dioxa-3H-perfluorononanoate	ADONA	$CF_3O(CF_2)_3OCFCF_2COONa$	E
6:2 Chlorinated polyfluorinated ether sulfonate	6:2Cl-PFESA	$Cl(CF_2)_6O(CF_2)_2SO_3^-$	E
8:2 Chlorinated perfluoroether ether sulfonate	8:2Cl-PFESA	$Cl(CF_2)_8O(CF_2)_2SO_3^-$	E
Perfluoro-methoxypropionic acid	PFMPA	$CF_3O(CF_2)_2COOH$	E

Compound name	Abbreviation	Structures	Category
Perfluoro-4-methoxybutanoic acid	PFMBA	$CF_3O(CF_2)_3COOH$	E
Perfluoro-3,6-dioxaheptanoic acid	NFDHA	$CF_3O(CF_2)_2OCF_2COOH$	E
Perfluoro-2-ethoxyethane sulfonate	PFEESA	$CF_3CF_2O(CF_2)_2SO_3^-$	E

Compounds were categorized as traditional (T) or emerging (E) using data presented in Xiao [1] and Wang et al. [2]

TableS2 Detection limit, quantitation limit and recovery of PFASs in different matrix

Compound	Water（n=6）			Soil/Sediment（n=6）			$PM_{2.5}$（n=6）		
	LLOD ng/L	LLOQ ng/L	Recovery %	LLOD ng/L	LLOQ ng/L	Recovery %	LLOD ng/L	LLOQ ng/L	Recovery %
PFBA	0.2	0.8	95.4±23.4	0.03	0.12	112.5±29.6	2.2	8.8	95.0±21.4
PFPeA	0.2	0.8	114.5±19.2	0.03	0.12	89.7±5.4	2.2	8.8	112.7±18
PFHxA	0.02	0.08	102.7±15.6	0.003	0.01	127.2±10.6	0.2	0.8	108.7±18
PFHpA	0.02	0.08	94.3±24.0	0.003	0.01	85.7±16.6	0.2	0.8	78.2±20.4
PFOA	0.02	0.08	131.5±35.2	0.003	0.01	97.9±28.6	0.2	0.8	130.3±34
PFNA	0.02	0.08	108.8±29.6	0.003	0.01	96±27.8	0.2	0.8	111.8±26.4
PFDA	0.04	0.2	84.9±20.2	0.004	0.02	98.4±4.6	0.3	1.2	81.0±30.0
PFUnA	0.03	0.2	102.7±22.4	0.004	0.02	75.4±18.8	0.3	1.2	94.4±30.0
PFDoA	0.03	0.2	91.7±18.6	0.004	0.02	72.4±29.6	0.3	1.2	105.8±0.6
PFTrA	0.03	0.2	102.6±38.2	0.004	0.02	104.9±1.0	0.3	1.2	81.6±28.2
PFTeA	0.03	0.2	80.3±34.2	0.004	0.02	108.2±25.2	0.3	1.2	111.0±1.0
PFBS	0.09	0.4	88.3±11.0	0.01	0.04	119.2±15.8	0.9	3.6	116.2±18.8
PFHxS	0.02	0.08	107.4±5.6	0.003	0.01	126.5±2.4	0.2	0.8	67.9±29.8
PFHpS	0.01	0.04	97.8±4.2	0.001	0.004	98.5±37.6	0.09	0.4	71.3±20.2
PFOS	0.01	0.04	108.6±11.6	0.001	0.004	99.9±26.4	0.09	0.4	106.5±7.8
PFDS	0.01	0.04	62.7±10.2	0.001	0.004	60.0±18.8	0.09	0.4	126.1±2.6
N-MeFOSAA	0.02	0.08	98±13.8	0.003	0.01	98.5±17.0	0.2	0.8	103.6±14.8
N-EtFOSAA	0.02	0.08	95.7±6.8	0.003	0.01	81.5±35.6	0.2	0.8	62.0±6.8
HFPO-DA	0.2	0.8	85±22.8	0.03	0.12	98.6±29.6	2.2	8.8	74.0±20.0
ADONA	0.005	0.02	120.6±10.0	0.001	0.004	121.2±18.2	0.04	0.2	116.5±8.8
6:2Cl-PFESA	0.004	0.02	75±37.2	0.001	0.004	63.7±20.2	0.04	0.2	104.7±3.8
8:2Cl-PFESA	0.005	0.02	110.3±19.4	0.001	0.004	112.0±14.4	0.04	0.2	82.6±34.0

Compound	Water（n=6）			Soil/Sediment（n=6）			$PM_{2.5}$（n=6）		
	LLOD ng/L	LLOQ ng/L	Recovery %	LLOD ng/L	LLOQ ng/L	Recovery %	LLOD ng/L	LLOQ ng/L	Recovery %
PFMPA	0.01	0.04	86.3±10.4	0.001	0.004	85.7±18.0	0.09	0.4	67.6±33.4
PFMBA	0.01	0.04	113.3±22.8	0.001	0.004	101.1±15.6	0.09	0.4	64.8±37.4
NFDHA	0.2	0.8	106.7±17.6	0.02	0.08	95.7±22.4	1.3	5.2	66.9±39.2
PFEESA	0.004	0.02	105.5±17.4	0.001	0.004	73.6±24.2	0.04	0.2	104.8±0.6

LLOD means detection limit; LLOQ means quantitation limit.

Table S3 Concentrations of 26 PFASs in the environment of Qiantang River watershed

Compound	Water (ng/L)			$PM_{2.5}$ (pg/m^3)			Soil (ng/g)			Sediment (ng/g)		
	Mean	Range	DF(%)	Mean	Range	DF(%)	Mean	Range	DF(%)	Mean	Range	DF(%)
PFBA	0.28	<LLOD~1.02	9.4	1.14	<LLOD~4.07	29	<LLOD	<LLOD	/	<LLOD	<LLOD	/
PFPeA	1.66	<LLOD~16.5	97	53.7	17.0~110.7	100	<LLOD	<LLOD	/	<LLOD	<LLOD	/
PFHxA	14.7	0.3~170	100	3.09	0.56~9.13	100	0.07	<LLOD~0.94	7.1	0.17	<LLOD~0.87	7.1
PFHpA	2.74	<LLOD~21.4	97	4.35	<LLOD~10.2	71	<LLOD	<LLOD	/	<LLOD	<LLOD	/
PFOA	96.7	3.01~525	100	50.4	9.01~216	100	1.05	0.71~2.8	100	1.18	0.72~3.00	100
PFNA	1.20	<LLOD~9.27	100	2.03	<LLOD~7.10	57	0.21	<LLOD~0.97	21	0.20	<LLOD~0.99	21
PFDA	2.21	<LLOD~4.98	94	1.59	<LLOD~7.66	71	0.61	<LLOD~1.22	50	0.26	<LLOD~1.29	50
PFUnA	0.23	<LLOD~3.16	59	2.30	<LLOD~10.3	71	0.38	<LLOD~1.33	29	0.53	<LLOD~1.35	29
PFDoA	0.46	<LLOD~1.46	41	3.91	<LLOD~13.7	29	<LLOD	<LLOD	7.1	<LLOD	<LLOD	/
PFTrA	0.06	<LLOD~0.84	19	5.85	<LLOD~19.9	86	<LLOD	<LLOD	/	<LLOD	<LLOD	/
PFTeA	0.04	<LLOD~0.51	16	6.26	<LLOD~19.7	71	0.12	<LLOD~1.68	/	<LLOD	<LLOD	7.1
PFBS	0.61	0.13~2.53	100	14.6	<LLOD~43.2	57	<LLOD	<LLOD	/	<LLOD	<LLOD	/
PFHxS	7.27	<LLOD~65.8	88	0.06	<LLOD~0.40	14	0.06	<LLOD~0.83	7.1	0.37	<LLOD~1.01	7.1
PFHpS	0.02	<LLOD~0.22	9.4	<LLOD	<LLOD	<LLOD	<LLOD	<LLOD	/	<LLOD	<LLOD	/
PFOS	2.31	<LLOD~12.3	97	6.30	2.90~17.0	100	0.57	<LLOD~1.05	57	0.60	<LLOD~1.02	57
PFDS	0.02	<LLOD~0.1	3.1	<LLOD	<LLOD	<LLOD	<LLOD	<LLOD	/	<LLOD	<LLOD	/
N-MeFOSAA	0.21	<LLOD~0.11	3.1	36.0	33.0~39.0	14	0.24	<LLOD~1.96	14	<LLOD	<LLOD	14
N-EtFOSAA	<LLOD	<LLOD	/	<LLOD	<LLOD	14	0.59	<LLOD~3.15	21	<LLOD	<LLOD	21
HFPO-DA	3.54	<LLOD~47.1	72	0.04	<LLOD~0.29	<LLOD	<LLOD	<LLOD	/	<LLOD	<LLOD	/
ADONA	0.09	<LLOD~1.27	28	0.07	<LLOD~0.48	43	<LLOD	<LLOD	/	<LLOD	<LLOD	/
6:2Cl-PFESA	34.1	<LLOD~4.61	91	<LLOD	<LLOD	57	0.57	<LLOD~1.16	50	0.68	<LLOD~1.14	50
8:2Cl-PFESA	2.16	<LLOD~0.34	16	0.04	<LLOD~0.24	<LLOD	0.23	<LLOD~1.63	14	0.33	<LLOD~1.63	14

Compound	Water (ng/L)			$PM_{2.5}$ (pg/m^3)			Soil (ng/g)			Sediment (ng/g)		
	Mean	Range	DF(%)	Mean	Range	DF(%)	Mean	Range	DF(%)	Mean	Range	DF(%)
NFDHA	1.13	<LLOD~5.07	78	0.59	<LLOD~2.73	<LLOD	<LLOD	<LLOD	/	<LLOD	<LLOD	/
PFEESA	0.03	<LLOD~0.49	9.4	<LLOD	<LLOD	<LLOD	<LLOD	<LLOD	/	<LLOD	<LLOD	/
PFMBA	0.03	<LLOD~0.46	13	<LLOD	<LLOD	<LLOD	<LLOD	<LLOD	/	<LLOD	<LLOD	/
Emerging PFASs	143	3.58~735	88	156	93.6~255	100	3.90	<LLOD	56	3.31	0.73~8.48	56
Traditional PFASs	6.08	<LLOD~51.4	100	0.63	<LLOD~2.77	25	0.81	0.72~11.1	25	1.00	<LLOD~2.76	25
ΣPFASs	149	3.58~786	100	156	93.9~255.2	77	4.70	<LLOD~2.79	46	4.31	0.73~8.48	46

LLOD means detection limit; DF means detection frequencies.

Table S4 The lgKd of PFASs between water and sediment / (ng/kg)

Compound	lgK_d
PFHxA	0.71
PFOA	1.16
PFNA	2.46
PFDA	2.85
PFUnA	4.02
PFHxS	2.36
PFOS	2.81
6:2Cl-PFESA	3.04
8:2Cl-PFESA	4.00

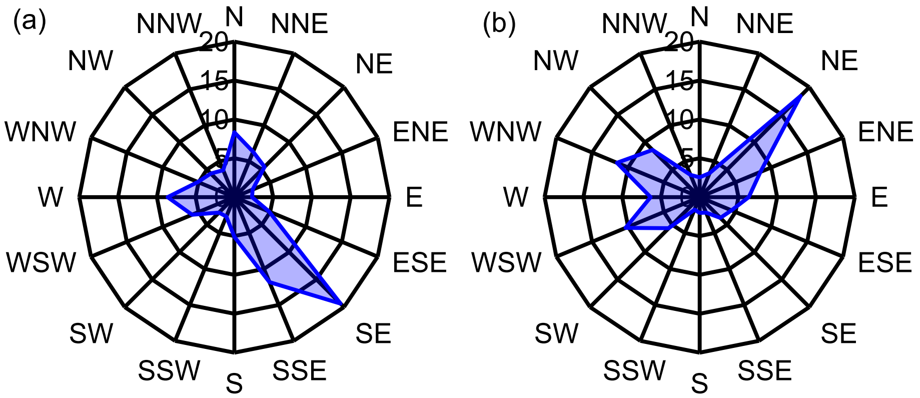

Fig. S1 The windrose diagram of (a) site 39 and (b) site 38

Reference

[1] F. Xiao, *Water Res*. 2017, 124, 482-495.

[2] Z. Y. Wang, I. T. Cousins, M. Scheringer, K. Hungerbuhler. *Environ Int*. 2013,60, 242-248.
